# AFM Analysis of Micron and Sub-Micron Sized Bridges Fabricated Using the Femtosecond Laser on YBCO Thin Films

**DOI:** 10.3390/mi11121088

**Published:** 2020-12-08

**Authors:** Patrice Umenne

**Affiliations:** Department of Electrical and Mining Engineering, University of South Africa, Florida 1709, South Africa; umennpo@unisa.ac.za; Tel.: +27-72-495-0922

**Keywords:** atomic force microscope, laser ablation diameter, separation distance (*S_W_*), sub-micron bridges, YBCO thin film

## Abstract

The research arose as a result of the need to use the femtosecond laser to fabricate sub-micron and nano-sized bridges that could be analyzed for the Josephson effect. The femtosecond laser has a low pulse duration of 130 femtoseconds. Hence in an optical setup it was assumed that it could prevent the thermal degradation of the superconductive material during fabrication. In this paper a series of micron and sub-micron sized bridges where fabricated on superconductive yttrium barium copper oxide (YBCO) thin film using the femtosecond laser, a spherical convex lens of focal length 30 mm and the G-code control programming language applied to a translation stage. The dimensions of the bridges fabricated where analyzed using the atomic force microscope (AFM). As a result, micron sized superconductive bridges of width 1.68 μm, 1.39 μm, 1.23 μm and sub-micron sized bridges of width 858 nm, 732 nm where fabricated. The length of this bridges ranged from 9.6 μm to 12.8 μm. The femtosecond laser technique and the spherical convex lens can be used to fabricate bridges in the sub-micron dimension.

## 1. Introduction

The main aim of this work was to fabricate sub-micron and nano sized bridges using the 775 nm wavelength femtosecond laser as it is used for ablative purposes in [1,2,3,4] on superconductive yttrium barium copper oxide (YBCO) thin film [5,6,7,8]. The resulting bridges could then be tested for the presence of the Josephson effect and used as Josephson junctions. In [9,10,11,12,13], several characteristics and applications of Josephson junctions are given in detail. The smaller the size of the bridges that are fabricated the more likely they are to show the Josephson effect. If proven to show the Josephson effect, they can then be used in applications such as superconducting quantum interference device (SQUID) as in [14] where they are used to detect the presence of magnetic moments. This papers focus is, however, restricted to several superconductive bridges fabricated in the achievable micron and sub-micron scale and their dimensional analysis using the atomic force microscope (AFM).

A series of bridges where fabricated using optical equipment such as reflective mirrors, iris diaphragm, spherical convex lens and optical techniques such as beam collimation and focusing to reduce the laser ablation diameter. Optimization techniques were used to reduce the laser ablation diameter by lowering the pulse energy of the laser [15] and to reduce the width of the bridge by reducing the distance between the laser ablation spots ($S_{w})$. In [15] several factors are discussed that can be used to control the minimum structure size of materials when machining with the femtosecond laser. G-code (RS-274) computer numerical control (CNC) programming language was used to enable the movement of the translation stage that holds the thin film sample. In the meantime, the laser was held in a stationary position while ablating the sample. In order to measure the dimensions of the bridges after fabrication the AFM [16,17,18,19,20] was utilized for imaging the sample. In [16,17,18,19,20], the AFM is utilized to scan and analyze, cells, molecules, superconductors, semiconductors and nanomaterials as examples to using the AFM. The AFM scans where done with type DT-NCHR diamond cantilever tips in tapping mode.

The main hypothesis in the research was the assumption that the low pulse duration (130 fs) of the femtosecond laser could reduce the thermal degradation of the superconductive YBCO thin film during ablation. This is required in the fabrication of Josephson junctions. However, a null hypothesis was achieved. Experiments showed that the pulse duration of the laser on the superconductive YBCO thin film during fabrication is actually a combined average of the feed rate of the translation stage which was set at 20 mm/min or 333 ${\mu \mathrm{ms}}^{-1}$, the frequency of the laser or pulse repetition rate in this case 1 kHz and the pulse duration of the laser which is 130 femtoseconds. As a result, the time spent by the laser on the YBCO sample during ablation is in fact much longer than femtoseconds. Hence some thermal degradation occurs. The main objective of the research was to use the femtosecond laser technique to fabricate micron, sub-micron and nano sized superconductive bridges on YBCO thin film that could be tested and used as Josephson junctions. The novelty of the research stems from the fact that the femtosecond laser has never been used previously to fabricate superconductive bridges on YBCO thin film that could be used as Josephson junctions. As a result, the effect of using the femtosecond laser for this purpose had not been previously analyzed.

## 2. Materials and Methods 

The YBCO thin films utilized for the experiment were procured from ceraco ceramic GmbH company. The thin films had the following specifications: 9 by 9 mm YBCO film, single sided 200 nm thickness, one side polished. The YBCO thin films came on either an ${\mathrm{LaAlO}}_{3}$ substrate or an MgO substrate. The thin films where the S-type smooth matrix useful for the manufacture of SQUIDS and were placed on a substrate of 10 by 10 by 0.5 mm dimension. The critical temperature of the YBCO thin film used was $T_{C}=87\;K$.

The laser beam was focused unto the YBCO sample to machine the bridges by using a spherical convex lens of 30 mm focal length. The femtosecond laser power ranged from (0–1000) mW. The laser was set at 2.1 mW in order to cut just slightly above the ablation threshold of the YBCO thin film and hence make the laser ablation diameter as small as possible. This optimization technique facilitates the fabrication of smaller bridges. The laser power setting of 2.1 mW in combination with the spherical convex lens of focal length 30 mm produced a laser ablation diameter of 15.8 μm.

**Figure 1:**
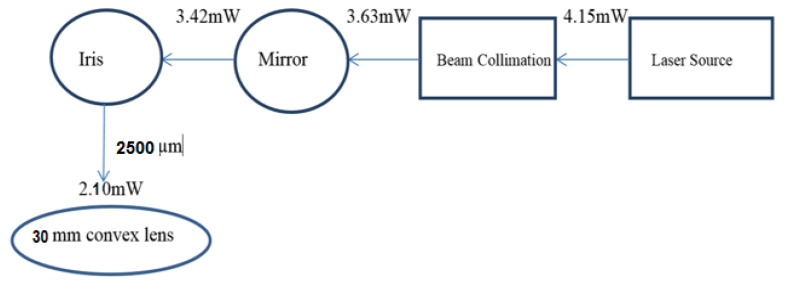
Block diagram of the overall laser optical experimental set up used to machine the bridges [21].

Figure 1 shows the block diagram of the optical set up used to optimize the laser ablation spot size and to fabricate the bridges with the femtosecond laser. The set up consists of the laser source, the beam collimation set up, reflective mirrors, iris (manually adjustable aperture) and the spherical convex lens of 30 mm focal length.

The function of the iris diaphragm is to remove unwanted sections of the laser in the outer periphery of the laser beam after the beam collimation process. The iris helps remove the poorly shaped sections of the laser beam produced by spherical aberration. This means only the central core of the laser beam would pass through to the focusing optics. The laser beam diameter from the laser source was 9.85 mm. The iris diaphragm could be adjusted from (0–10,000) μm. The iris was set to a diameter of 2500 μm, thus reducing the laser beam diameter from 9.85 to 2.5 mm. The laser beam is then passed through the focusing optics and a laser ablation diameter of 15.8 μm is produced. During fabrication the YBCO sample is placed on a translation stage whose movement is programmed using G-code. The laser was kept stationary while the translation stage moved during the fabrication of the bridges.

The width of the bridges after fabrication is defined by the formula in Equation (1) [21]:

(1) $$width\;of\;bridge=\;S_{W}-laser\;ablation\;diameter$$

where $S_{W}$ is the distance between the laser ablation spots along the length of the square sample.

The length of the bridges is defined by the formula in Equation (2):

(2) $$Length\;of\;bridge=\;\left|S_{L}-Laser\;ablation\;diameter\right|$$

where $S_{L}$ is the distance between the laser ablation spots along the width of the square sample.

By bringing the laser ablation spots closer along the length of the sample the width of the bridge can be controlled. Similarly, by moving the laser ablation spots along the width of the sample you can control the length of the bridge fabricated. Initially the ablation strips are etched on the sample. The function of the ablation strips is to separate one bridge from another electronically. The ablation strip lines are fabricated by moving the laser vertically along the length of the YBCO square film. When the ablation strips are completed, they will have a width of 0.5 mm. The laser is moved vertically down the length of the YBCO thin film according to a specific factor. Horizontally into the sample, out of the sample and then vertically down by a factor. Across the width of the YBCO thin film, up along the length determined by a factor, again horizontally into the sample, back out and then back to the top along the length determined by a specific factor. This movement fabricates a bridge that is S-shaped and whose images are shown in the result section.

## 3. Results

Table 1 summarizes the bridges fabricated, the distance between the laser ablation diameters $S_{W}$ set in the program, the laser ablation diameter and the width of the bridge that was machined. During the fabrication of all these bridges a conventional spherical convex lens of focal length 30 mm was used as the focusing optics.

### 3.1. AFM Analysis of Micro-A

**Figure 2:**
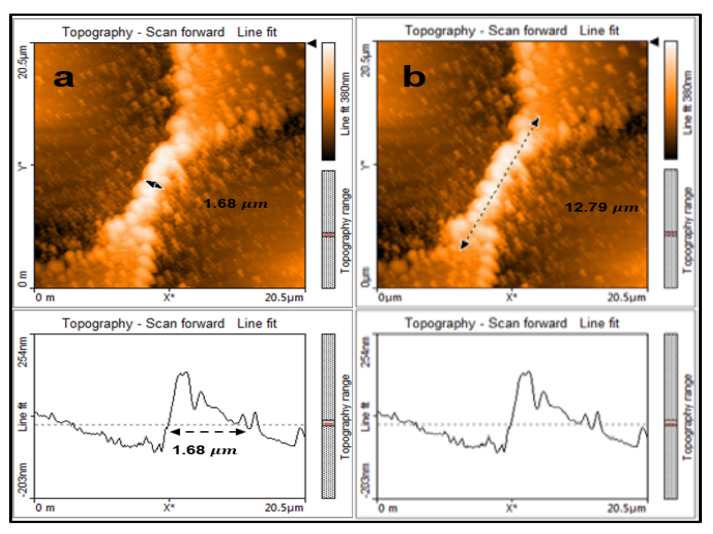
(**a**) topography line fit shows the width of the bridge at 1.68 μm and (**b**) topography line fit shows the length of the bridge at 12.79 μm.

When fabricating the bridge Micro-A, the distance between the laser ablation spots ($S_{W}$) along the length of the square sample was set at 18 μm. This setting determines the width of the bridge. The distance between the laser ablation spots ($S_{L}$) along the width of the square sample was set at approximately 5 μm. This setting determines the length of the bridge. The laser ablation diameter using the 30 mm focal length convex lens was 15.8 μm. As a result, a bridge of width 1.68 μm and length 12.79 μm was achieved as can be seen in the panel Figure 2. We focus on a very small scan area on the sample that is 20.5 by 20.5 μm.

The measurements are taken using the AFM as the cantilever tip scans transversely at 90 degrees to the laser ablation spot. By using the topography line fit shown in panel Figure 2a the width of the bridge is presented as an amplitude above the zero axis. The width of the amplitude shown in between the black arrows in the figure is 1.68 μm.

### 3.2. AFM Analysis of Micro-B

**Figure 3:**
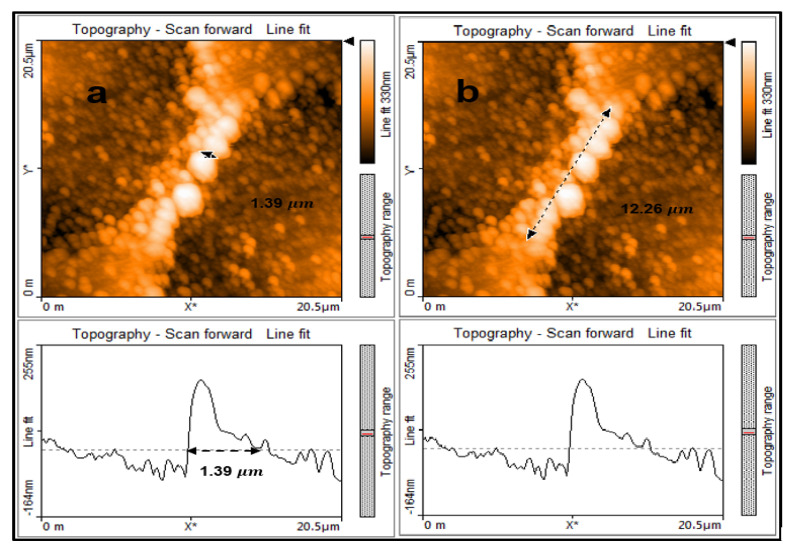
(**a**) topography line fit shows the width of the bridge at 1.39 μm and (**b**) topography line fit shows the length of the bridge at 12.26 μm.

In the bridge Micro-B, the distance between the laser ablation spots ($S_{W}$) along the length of the square sample was set at 17.5 μm. The distance between the laser ablation spots ($S_{L}$) along the width of the sample was set at 5 μm, just as for Micro-A. The laser ablation diameter remained the same as 15.8 μm. A bridge of width 1.39 μm and length 12.26 μm was fabricated as can be seen in panel Figure 3. Again, the scan area was set at 20.5 by 20.5 μm. Therefore, by reducing the distance between the laser ablation spots ($S_{W}$) and keeping the laser ablation diameter constant one can produce a bridge of smaller width as per Equation (1).

The measurements were taken with an AFM. In the topography line fit in panel Figure 3a the width of the bridge is presented as an amplitude above the zero axis. The width of the amplitude in between the black arrows in the figure is 1.39 μm.

### 3.3. AFM Analysis of Micro-C

**Figure 4:**
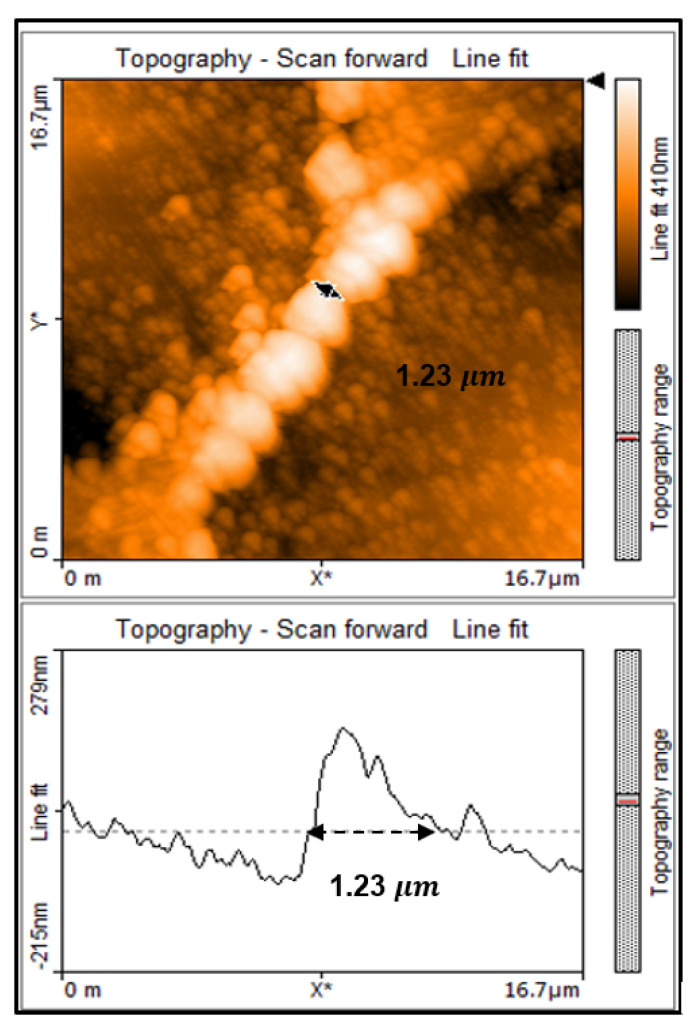
Topography line fit shows the width of the bridge at 1.23 μm.

In the bridge Micro-C, the distance between the laser ablation spots ($S_{W}$) along the length of the square sample was set at 16.5 μm. The distance between the laser ablation spots ($S_{L}$) along the width of the sample was set at 5 μm, just as for Micro-A. The laser ablation diameter remained the same as 15.8 μm. Using the theory of Equation (1), a bridge of width 700 nm, is expected however, a bridge of width 1.23 μm is achieved as can be seen in Figure 4.

In order to determine the exact width of this bridge the scan area was reduced to 16.7 by 16.7 μm.

### 3.4. AFM Analysis of SubMicro-D

**Figure 5:**
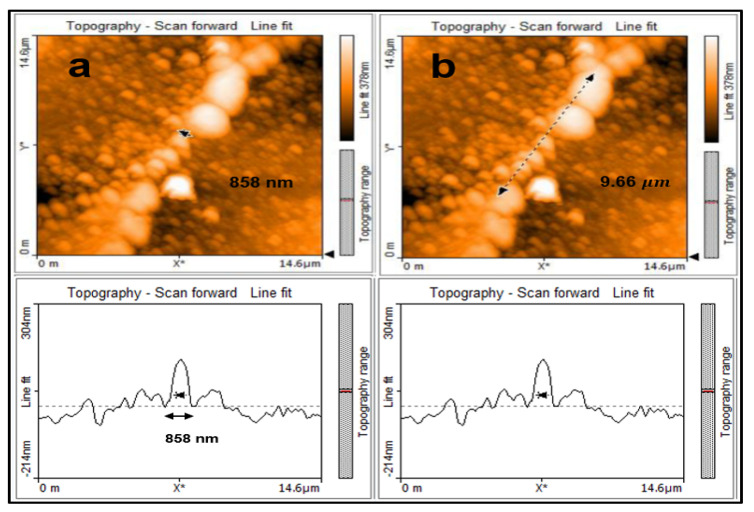
(**a**) topography line fit shows the width of the bridge at 858 nm and (**b**) topography line fit shows the length of the bridge at 9.66 μm.

For the bridge SubMicro-D, the distance between the laser ablation spots ($S_{W}$) along the length of the square sample was set at 16.5 μm. The distance between the laser ablation spots ($S_{L}$) along the width of the sample was set at 5 μm, just as for Micro-A. As a result, a bridge of width 858 nm and length 9.66 μm was fabricated as can be seen in the panel Figure 5.

In order to establish the dimensions of the bridge SubMicro-D the scan area was reduced to 14.6 by 14.6 μm.

### 3.5. AFM Analysis of SubMicro-E

**Figure 6:**
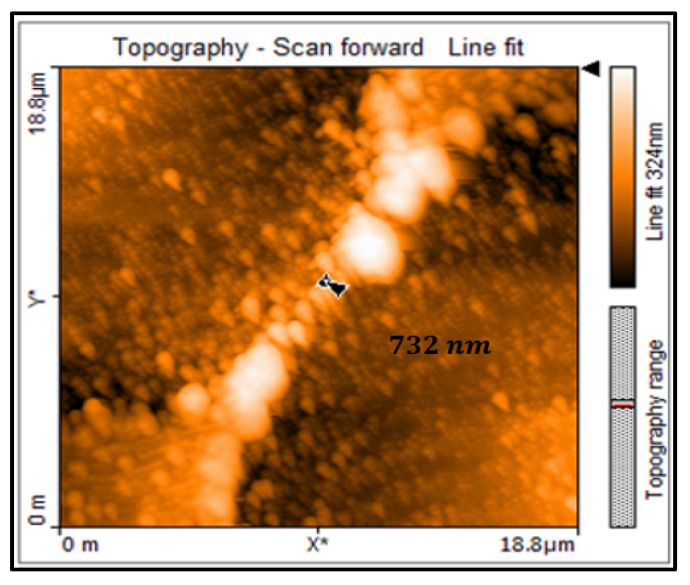
Topography line fit shows the width of the bridge at 732 nm.

In the design of the bridge SubMicro-E, the distance between the laser ablation spots ($S_{W}$) along the length of the square sample was set at 16 μm. The distance between the laser ablation spots ($S_{L}$) along the width of the sample was set at 5 μm, just as for Micro-A. A bridge of width 732 nm was achieved as can be seen in Figure 6. The scan area was set at 18.8 by 18.8 μm. The figure shows that if we machine any narrower the bridge can collapse. The reason is because we are approaching the diffraction limit of fabricating small structures with the femtosecond laser, which has a wavelength of 775 nm. In addition, in Figure 6 it can be seen that there is some thermal degradation on the YBCO thin film. This is shown by the different phases of ablation close to the bridge. There is a dark phase where there is more heating closer to the bridge and a lighter phase where there is less heating away from the bridge in the diameter of the laser ablation spot.

## 4. Current-Voltage Characteristics (IVC’s) of the Bridges

**Figure 7:**
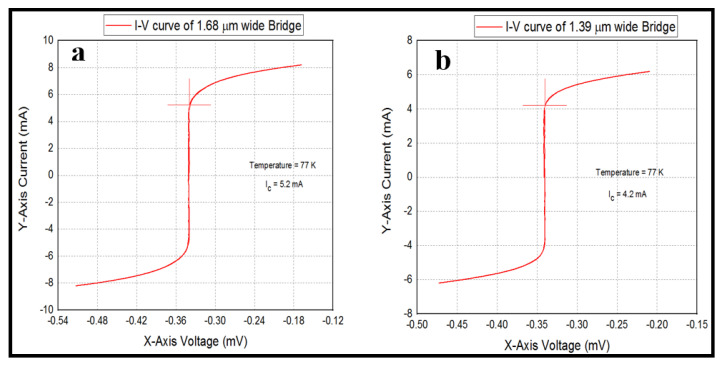
(**a**) I-V curve for Micro-A bridge and (**b**) I-V curve for Micro-B bridge.

The I-V characteristic curves for the superconductive bridges Micro-A and Micro-B can be seen in Figure 7. Micro-A has a critical current $I_{C}$ of 5.2 mA at temperature of 77 K and Micro-B has a critical current $I_{C}\;$ of 4.2 mA at a temperature of 77 K. The critical current $I_{C}$ for the superconductive bridge Micro-A slightly exceeds that of Micro-B. This can be explained by the fact that Micro-A is wider and longer than Micro-B, hence it is able to pass more current than Micro-B. Furthermore, from the AFM scans of Micro-A and Micro-B the geometrical boundaries of Micro-A are slightly better defined than that of Micro-B. This would mean that the superconductive phase of Micro-A is less damaged by the femtosecond laser during fabrication, hence produces more superconductive current at a temperature of 77 K. Critical currents $I_{C}$ in the mA range are standard values of current at this temperature for this bridge dimensions.

## 5. Discussion

At the beginning of the research a hypothesis was made, that by using the femtosecond laser to machine the superconductive bridges thermal degradation of the superconductive sample could be reduced, since the femtosecond laser has a low pulse duration of 130 femtoseconds. However, a null hypothesis was achieved because the average time the laser spends on the sample depends not only on the pulse duration of the laser (130 fs), but also on the frequency of the laser (1 kHz) and the translation stage feed-rate (20 mm/min). Thermal degradation of the sample is represented by the dark phases in Figure 6 close to the bridge where there is more heating from the laser. Further away from the bridge there is a light phase indicative of less heating from laser and less thermal damage. When the YBCO thin film is less damaged by the heat from the laser it maintains a light phase seen on the AFM scans. The effect of the thermal degradation on the YBCO thin film is that the material changes from a superconductive phase to a non-superconductive phase that is either a resistor or an electronically open material. In the non-superconductive phase, it is impossible to produce a Josephson junction which was the original objective of the research. All the bridges fabricated in this research have an intact superconductive phase and could possibly be used as Josephson junctions. This is indicated in the I-V curve characteristics. The femtosecond laser technique was used instead of micro-milling techniques since micro-milling techniques require contact and use friction which produces heat and requires work to be done at temperatures that do not exceed 35 °C. Such temperatures would easily damage the superconductive YBCO material which is sensitive to high temperatures and can operate at a temperature that does not exceed 87 K (−186 °C). Moreover, the superconductive YBCO material requires non-contact techniques during fabrication while micro-milling is a contact method. Finally, the micro-milling technique generally can fabricate only micron sized dimensions while sub-micron and nano-scales are necessary to see the Josephson effect. The femtosecond laser technique is non-contact, can fabricate very small structures and generally has a very low-pulse duration on average that can evade heating of the sample.

## 6. Conclusions

In conclusion three micron-sized bridges; Micron-A, Micron-B, Micron-C and two sub-micron sized bridges; SubMicron-D and SubMicron-E were fabricated on superconductive YBCO thin film for possible use as a Josephson junction. These bridges were analyzed for their dimensions with the aid of the AFM. In the end it was discovered that the smaller the distance between the laser ablation spots $S_{W}$ along the length of the square sample, the smaller the width of the bridge that is fabricated. This is the case if the laser ablation diameter is kept constant. There is however a limitation on the smallest width of the bridge that can be fabricated which will depend on the wavelength of the laser. Furthermore, a certain amount of thermal degradation occurs on the YBCO bridge even when using the femtosecond laser with a low pulse duration.

## Figures and Tables

**Table 1:** Summary of bridges fabricated and their dimensions.

Number	Name of Bridge	Separation Distance between Laser Ablation Spots (*Sw*)	Laser Ablation Diameter	Width of Bridge Formed
1	Micro-A	18 μm	15.8 μm	1.68 μm
2	Micro-B	17.5 μm	15.8 μm	1.39 μm
3	Micro-C	16.5 μm	15.8 μm	1.23 μm
4	SubMicro-D	16.5 μm	15.8 μm	858 nm
5	SubMicro-E	16 μm	15.8 μm	732 nm
